# ASGE Endoscopy Live: interventional EUS and endohepatology. A one-stop shop endohepatology case of a patient with cirrhosis of unknown etiology

**DOI:** 10.1016/j.igie.2023.07.018

**Published:** 2023-08-04

**Authors:** Pichamol Jirapinyo, Kenneth J. Chang

**Affiliations:** 1 Division of Gastroenterology, Hepatology and Endoscopy, Brigham and Women’s Hospital, Boston, Massachusetts, USA; 2 Harvard Medical School, Boston, Massachusetts, USA; 3 H.H. Chao Comprehensive Digestive Disease Center, University of California, Irvine Medical Center, Orange, California, USA

The American Society for Gastrointestinal Endoscopy (ASGE) has organized multiple live endoscopy courses with a focus on different aspects of diagnostic and therapeutic endoscopy. The “ASGE Endoscopy Live: interventional EUS and endohepatology” course took place on July 13, 2023 with 9 live interventional EUS cases broadcasted from 6 different centers (5 U.S. centers and 1 European center). Table 1 summarizes the cases and outcomes.

**Table 1:** Summary of cases performed during the American Society for Gastrointestinal Endoscopy Endoscopy Live: interventional EUS and endohepatology course on July 13, 2023.

Case no.	Procedure	Age (y) Sex	Indication	Technical outcome	Adverse events
1	EUS-guided PFC drainage	70 Female	An 11-cm PFC in the pancreas body secondary to gallstone pancreatitis	Successful drainage of the PFC with a 20-mm LAMS	None
2	EUS-guided GE	83 Female	Malignant gastric outlet obstruction secondary to metastatic pancreatic adenocarcinoma	Successful GE performed with a 15-mm LAMS	None
3	EUS-guided hepaticogastrostomy	56 Male	A 12-cm hepatic mass with dilated left and right intrahepatic ducts and elevated bilirubin; status after placement of a 10F plastic stent into the right hepatic duct	Successful deployment of an 8-mm × 8-cm partially covered metal stent from the common hepatic duct into the stomach	None
4	EUS-guided SWE EUS-guided PPG measurement EUS-guided LB	75 Male	Concern for cirrhosis of unclear etiology based on fibrosis-4 index of 3.61 and liver stiffness measurement on vibration-controlled transient elastography of 22 kPa	Successful SWE, PPG measurement, and LB performed, all of which confirmed cirrhosis with portal hypertension	None
5	EUS-guided GE	82 Male	Malignant duodenal obstruction secondary to ampullary adenocarcinoma	Successful GE performed with a 20-mm LAMS	None
6	EUS-guided gallbladder drainage	55 Male	Symptomatic cholelithiasis in a patient who had s/p myocardial infarction, cardiogenic shock, and ischemic bowel s/p small-bowel resection and colectomy	Successful transduodenal drainage of the gallbladder with a 10-mm LAMS with a double-pigtail plastic stent within the LAMS	None
7	EUS-directed transgastric ERCP	47 Female	Choledocholithiasis in a patient with a Roux-en-Y gastric bypass anatomy	Successful single-session EUS-directed transgastric ERCP with a 20-mm LAMS followed by ERCP through the LAMS	None
8	EUS-guided GE and EUS-guided pancreaticogastrostomy	56 Male	Malignant duodenal obstruction secondary to pancreatic head adenocarcinoma and pain secondary to pancreatic duct obstruction	Successful pancreaticogastrostomy with a 7F × 9-cm pancreatic duct stent; successful GE performed with a 20-mm LAMS	None
9	EUS-guided gastric varices coiling with injection of absorbable gelatin sponge	48 Female	Anemia secondary to intermittently oozing gastric varices (type 2 gastroesophageal varices) in a patient with decompensated cirrhosis secondary to alcohol use	Successful gastric varices obliteration with placement of four .035-inch × 18-mm × 20-cm coils followed by injection of absorbable gelatin sponge	None

*PFC*, Pancreatic fluid collection; *LAMS*, lumen-apposing metal stent; *GE*, gastroenterostomy; *SWE*, shear-wave elastography; *PPG*, portosystemic pressure gradient; *LB*, liver biopsy sampling; *s/p*, status post.

## Case Presentation

A 75-year-old man with class II obesity, hypertension, Crohn’s disease (in remission), and status postcholecystectomy presented with asymptomatic jaundice. He was previously informed that he had “fatty liver.” His medications included irbesartan 150 mg daily and mercaptopurine 50 mg daily. He denied alcohol or illicit drug use, smoking, or tattooing. On examination, his body mass index was 35.6 kg/m^2^. His laboratory values were aspartate aminotransferase of 34 U/L, alanine aminotransferase of 31 U/L, total bilirubin of 6.8 mg/dL, direct bilirubin of .8 mg/dL, and alkaline phosphatase of 107 U/L. His albumin was 4.2 g/dL, international normalized ratio of 1.3, and platelet counts of 127,000/μL. The workup to assess for an etiology of his chronic liver disease otherwise was unrevealing. On vibration-controlled transient elastography, his liver stiffness measurement was 22 kPa, suggestive of F4 fibrosis/cirrhosis. His abdominal magnetic resonance imaging revealed a diffusely nodular liver with no mass, moderate splenomegaly of 16 cm, and perisplenic vessels. He was referred for endohepatology procedures to diagnose the etiology of and risk stratify the stage of his chronic liver disease.

## Brief Description of the Live Endoscopy Video

The concept of a one-stop shop endohepatology workup was demonstrated during this ASGE Live Endoscopy Course. Specifically, the patient underwent an outpatient EUS where 3 endohepatology procedures were performed in 1 session: EUS-guided shear-wave elastography measurement (EUS-SWE), EUS-guided portosystemic pressure gradient (EUS-PPG) measurement, and EUS-guided liver biopsy sampling (EUS-LB) (Video 1, available online at www.igiejournal.org).

## Procedural and Clinical Outcomes

A single-session endohepatology workup was accomplished with 100% technical success. First, a diagnostic EUS was performed using a linear echoendoscope (GF-UT180; Olympus, Central Valley, Pa, USA) and US processor (Aloka Arietta 850; Olympus). The liver parenchyma appeared coarse and nodular with a blunted liver edge. An EUS-SWE was then performed. The mean SWE of the left side of the liver, right side of the liver, and spleen was 22.4 kPa, 24.6 kPa, and 44.9 kPa, respectively, which suggested the patient had cirrhosis. Subsequently, EUS-PPG measurement was achieved using the EchoTip Insight kit (Cook Medical, Bloomington, Ind, USA). The mean hepatic venous pressure was 7 mm Hg, and the mean portal venous pressure was 16 mm Hg. Therefore, his PPG measurement was 9 mm Hg, suggestive of the presence of portal hypertension without clinically significant portal hypertension. Finally, EUS-guided LB of the right hepatic lobe was performed using a 19-gauge Acquire fine-needle biopsy needle (Boston Scientific, Marlborough, Mass, USA). One pass with 4 actuations was performed, which resulted in a 27-cm total specimen length. There were no procedure-related adverse events. The patient was discharged home on the same day in a stable condition. The pathology of the LB showed mild steatosis with moderately active steatohepatitis and cirrhosis. Trichrome stain showed bridging fibrosis (stage 4/4).

## Technical Highlights

1. The procedure is best performed with the patient under general anesthesia and in a supine position.
2. The EUS-SWE procedure:
  2.1. Avoid applying too much pressure on the echoendoscope probe because this may result in a falsely high liver stiffness measurement.
  2.2. Ensure that VsN—the percentage of the net effective shear-wave velocity, which represents a reliability index of shear-wave measurement—is greater than 50% for each shear-wave measurement.
  2.3 The SWE of the spleen of greater than 25 kPa appears to be associated with portal hypertension.
3. The EUS-PPG measurement procedure:
  3.1. Place and zero the manometer at the patient’s midaxillary level. The noncompressible tube and needle are flushed with heparinized saline solution. Attention should be paid to remove any air bubble within this closed system.
  3.2. During EUS-PPG measurement, the most common sites for pressure measurement are the middle hepatic vein and the umbilical portion of the left portal vein.
4. The EUS-LB procedure:
  4.1. A 19-gauge fine-needle biopsy needle is preferred to a 19-gauge FNA needle or a 22-gauge fine-needle biopsy needle.
  4.2. A wet-suction technique provides higher tissue yields compared with the dry-suction technique. During the wet-suction technique, the needle stylet is removed, and the needle is flushed with a heparin flush. The suction syringe is filled with 3 to 4 mL of heparin, the stopcock is turned to off, and then the suction syringe is set at a full suction setting.
  4.3. For the biopsy sampling technique, the needle is first introduced about 1 cm into the liver parenchyma through the gastric or duodenal wall. We will call this position the starting position. The suction syringe is then removed from the needle to allow the needle pressure to equilibrate with room air. The needle is then advanced into the liver parenchyma. The needle stroke should be of high velocity, and the needle course should be at least 3 cm without any vessels. Once the needle is deep within the liver parenchyma, the suction syringe is placed on the needle and the stopcock is turned on for 5 seconds and then off. The needle is withdrawn back to the starting position, and the suction syringe is removed from the needle. The needle is then readvanced into the liver parenchyma, and the same steps are repeated for a total of 2 to 4 actuations. The last needle withdrawal should be performed under Doppler US to ensure there is no bleeding within the needle track.
  4.4. On needle withdrawal, if Doppler US shows an active flow concerning for bleeding within the needle tract, try changing the needle angle. If Doppler US continues to flow, remove the suction syringe from the needle and put the stylet back in within the needle. As you advance the stylet in approximately 40% to 50% of the needle length, some of the liver core samples will be pushed back into the liver parenchyma to cause a tamponade. Because the stylet is only advanced in partially, one should be able to retrieve the remainder of the liver core samples.

## Conclusion

This endohepatology case demonstrates the technical aspects of a single-session EUS-SWE, EUS-PPG measurement, and EUS-LB. Multiple practical tips and tricks are summarized in the video and text.

## Patient Consent

The authors have received appropriate patient consent for the publication of this article.

## Disclosure

The following authors disclosed financial relationships: P. Jirapinyo: Consultant for Apollo Endosurgery, Erbe, GI Dynamics, and Spatz Medical; research support from Apollo Endosurgery, Boston Scientific, Fractyl, GI Dynamics, and USGI Medical; advisory board for Apollo Endosurgery; royalties from Endosim. K. J. Chang: Consultant for Cook Medical, Olympus, Medtronic, and Boston Scientific.

## Supplementary data

Video 1 EUS-guided shear-wave elastography measurement, EUS-guided portosystemic pressure gradient measurement, and EUS-guided liver biopsy sampling performed during the ASGE Endoscopy Live: interventional EUS and endohepatology course on July 13, 2023.

